# The effect of impedance to root growth on plant architecture in wheat

**DOI:** 10.1007/s11104-015-2462-0

**Published:** 2015-04-11

**Authors:** Kemo Jin, Jianbo Shen, Rhys W. Ashton, Rodger P. White, Ian C. Dodd, Andrew L. Phillips, Martin A. J. Parry, William R. Whalley

**Affiliations:** Department of Plant Nutrition, College of Resource and Environmental Sciences, China Agricultural University, Beijing, 100193 China; Rothamsted Research, West Common, Harpenden St Albans, AL5 2JQ UK; The Lancaster Environment Centre, Lancaster University, Lancaster, LA1 4YQ UK

**Keywords:** Root impedance, Leaf elongation, Root growth angle, Rht alleles

## Abstract

**Background and aims:**

We were interested in the effect of impedance to root growth on root and shoot architecture of wheat. It is known that *Rht-1* semi-dwarfing alleles decrease the degree of leaf stunting due to root impedance. We compared commercial wheat cultivars containing different *Rht-1* alleles to determine whether leaf stunting caused by root impedance differed between cultivars. We investigated effects of impedance to root growth on the angular spread of roots.

**Methods:**

The wheat cultivars Avalon, Robigus and Battalion, carrying semi-dwarfing alleles of *Rht-1*, and cv. Cadenza, carrying the tall, wild-type allele, were grown under two levels of soil strength in a sand culture system designed to allow the mechanical impedance of the root growth environment to be adjusted independently of water and nutrient availability.

**Results:**

Impeded roots grew more steeply than non-impeded roots: the angular spread of roots decreased from 55° to 43° from the vertical, but the genotypic effects were weak. Root impedance reduced leaf elongation and the number of tillers. Leaf area and total root length provided a common relationship across all genotype x treatment combinations. Leaf stunting in Cadenza was more severe.

**Conclusion:**

Our data support the hypothesis that the severity of leaf stunting due to root impedance is related to the *Rht* allele. Impeded roots had a smaller angular spread.

## Introduction

To be productive wheat needs uninhibited shoot growth supported by a root system that is efficient at capturing available water and nutrients. Unfortunately, abiotic stresses in drying soil stunt both shoot and root growth (Masle and Passioura 1987). The early effects of soil drying are thought to be related to (1) decreased root elongation due to the direct effect of strong soil that is more difficult to penetrate and (2) the stunting of shoot growth by the indirect effect of chemical root-to-shoot signalling within the plant. The stunting effect of root impedance on leaf elongation is well reported (Masle and Passioura 1987; Jin et al. 2013) but very little is known about the extent of any genotypic variation. Three near-isogenic lines (NILs) in cvs. Mercia and April Bearded (containing *Rht-B1a*, *Rht-B1b* or *Rht-B1c*) responded differently to root impedance (Coelho Filho et al. 2013). In the gibberellin-insensitive (GA-insensitive) severe dwarf NIL containing the *Rht-b1c* allele, leaf elongation was not reduced by root impedance, whereas leaf elongation in the tall *Rht-B1a* and semi dwarf *Rht-B1b*lines was reduced. In comparison with the tall *Rht-B1a*, leaf stunting in the semi dwarf *Rht-B1b* was proportionately smaller. Although reduced GA signalling may be implicated in stunting leaf growth due to the root impedance, very little is known about the genotypic variability in leaf stunting in commercial wheat cultivars.

The importance of root system architecture for maintaining crop yield under water limited agriculture, associated with increased mechanical impedance, is becoming recognized and is of increasing interest to plant breeders (Ho et al. 2005; Gewin 2010; Mi et al. 2010; Lynch 2013; White et al. 2013; Chen et al. 2014; Rebetzke et al. 2014). Increasing root system access to water deep in the soil profile may be a promising way to increase water capture under water limiting conditions (Dodd et al. 2011; Trachsel et al. 2013; White et al. 2013; Jin et al. 2013). A narrower angular spread of roots is associated with deeper rooting (Lynch 2013; Trachsel et al. 2013; White et al. 2013). The angle of incidence of a root at a strong soil layer has a large effect on the probability of root penetration and a near vertical root is far more likely to penetrate a horizontal layer of strong soil (Jin et al. 2013). Thus the increased likelihood of penetration of a horizontal layer by a near vertical root is consistent with deeper rooting. Whalley et al. (2013) found that the only genotypic variation in root penetration ability in wheat was found in near vertical roots.

Plants with a narrower angular spread of roots are thought to be at an advantage in water limited environments (Manschadi et al. 2006, 2008) whereas a wide angular spread of roots is thought to benefit nutrient uptake, especially P (Ge et al. 2000; Rubio et al. 2001; Lynch 2007, 2011; Shen et al. 2013). As discussed previously, steep roots are better at penetrating strong interfaces in the soil (Dexter and Hewitt 1978; Whalley et al. 2013). Thus for a cultivar with much steeper roots there will be a greatly increased probability that more roots can penetrate any possible strong layer, which tend to be ubiquitous in all mineral soils, both natural and cultivated (Whalley et al. 2013). The angular spread of roots is determined by the gravitropic response of the elongating roots. From experiments in gel chambers, it is known that genotypic variability in the angular spread of barley roots exists in young seedlings (Bengough et al. 2004) as well as in wheat grown to maturity in soil (Manschadi et al. 2006). The interaction between gravitropism, abiotic stress and the penetration of strong layers seems to be a neglected area, although considerable progress has been made with respect to the transmission of a sensed gravitropic response (from the root cap to the root elongation zones) and the subsequent interpretation of modified growth patterns (Boonsirichai et al. 2002; Band et al. 2012; Toyota and Gilroy 2013).

Genotypic differences in the gravitropic response of two different wheat cultivars (Oyanagi et al. 1992) have been found. In one of the wheat cultivars, the root orientation became more vertical at water potentials smaller than -50 kPa, while in the other wheat cultivar a near vertical root growth habit was independent of external water potential. Similar data does not exist for the effects of soil strength on angular spread, although at a matric potential of -50 kPa, soil strength is likely to impede root elongation (Bengough and Mullins 1991; Whalley et al. 2006, 2007). Increased mechanical impedance in soil, simulated in this work by using sand cultures, is one of the first effects of soil drying (To and Kay 2005; Bengough et al. 2006; Whalley et al. 2007) and decreases yield of field grown crops (Whalley et al. 2006, 2008)

There has been limited work in evaluating the root architecture of UK wheat cultivars, even though soil drying can limit crop yields (Whalley et al. 2006; Dodd et al. 2011). Under optimal soil conditions, Battalion has steeper root growth angles than Robigus (Whalley et al 2013). It is widely reported that semi dwarf wheat cultivars tend to perform better than tall wheat cultivars when water and nutrient availability is optimal, but tall, GA-responsive varieties tend to be more resilient to adverse conditions (Butler et al. 2005). In this study we explore the effect of strong soil on root and shoot architecture because both are implicated in the plasticity of the response of wheat to adverse conditions. We studied the effects of root impedance because increases in soil strength are often the first effects of water shortage in the soil. Leaf stunting and steeper root growth would be a helpful adaptive response to minimize water use and increase the water availability respectively.

## Materials and methods

### Plant material

In this study, we used Avalon and Cadenza wheat cultivars because they are parent lines of a mapping population but they also differ in their dwarfing alleles (Avalon *Rht-D1b* semi-dwarfing allele; Cadenza *Rht-D1a/B1a* tall). Robigus and Battalion, commercial semi-dwarf winter wheat cultivars containing the *Rht-B1b* and *Rht-D1b* alleles, respectively, were also used in this work. Previous work (Whalley et al. 2013) suggested that the angular growth of roots in Robigus and Battalion differed when grown under low mechanical impedance. Seeds were germinated between two sheets of wet filter paper in Petri dishes which were wrapped in black nylon fabric to exclude light. Two-day old seedlings were planted into the sand cores described below.

### Experimental approach

**Fig. 1:**
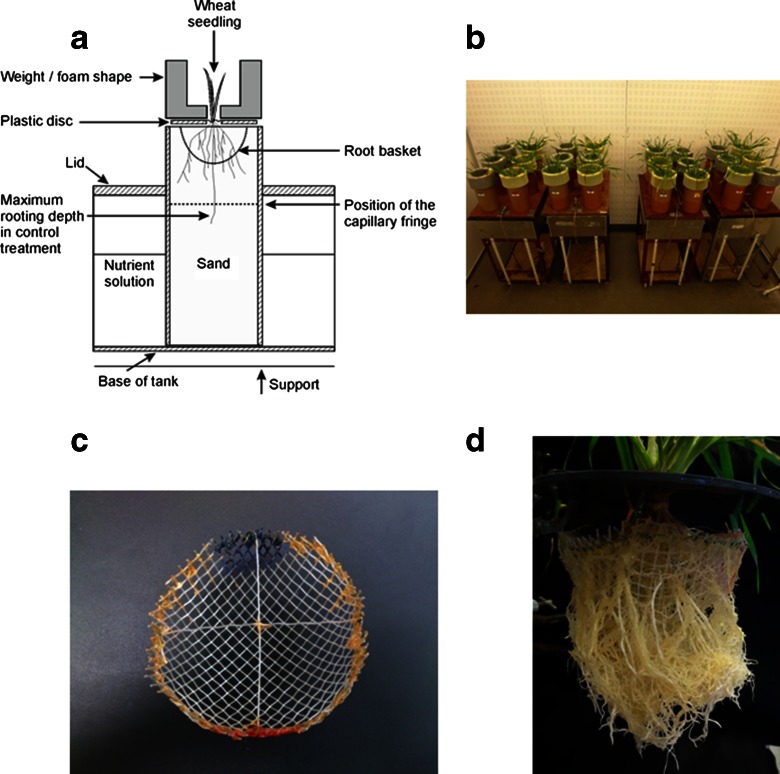
A schematic representation of the experimental growth system (**a**), which shows the position of the capillary fringe to scale. Below the capillary fringe the sand is saturated. The length of the pots is 45 cm. The system in use is shown in the photograph (**b**). The hemispherical mesh basket (**c**) which was used to allow root growth angle to be recorded at harvest is in photograph (**d**). The size of the basket is 10 cm in diameter and 5 cm in depth. The cross on the top of the basket helped to keep the plant in the correct position at harvest

To investigate the effects of strong soil in isolation of other abiotic stresses we used the sand culture system. This has been previously described in the context of crop emergence studies (Whalley et al. 1999) and to study the effects of high mechanical impedance on the growth of rice (Clark et al. 2002) and wheat (Whalley et al. 2006; Coelho Filho et al. 2013). The sand-culture system (Coelho Filho et al. 2013) allows mechanical impedance to be varied independently of aeration and water status of the growing medium. When a weight is placed on the sand surface, the mechanical impedance of the medium is increased as the resistance of sand grains to displacement is increased, but there is negligible compaction of the sand. In this work the experimental approach was modified by burying a hemispherical basket, 10 cm in diameter, in the centre of the tube, under the surface of the sand (see Fig. 1). The basket was constructed of mesh with holes 5x5 mm in size.

We used rigid plastic tubes 45 cm long and 15 cm in diameter. Before planting the wheat seedling, to achieve high impedance, a 17 kg weight was placed on the surface of the sand (Redhill T grade silica sand, Sibelco UK, CW1 14TF, UK) and its weight was evenly distributed by placing it on a plastic disc 14 cm in diameter. This produced a penetrometer resistance of approximately 0.75 MPa (Whalley et al. 1999). The low impedance control treatment had a foam object of the same shape as the steel weight and the penetrometer resistance in this treatment was approximately 0.19 MPa. The same type of sand and packing method was used here as described by Whalley et al. (1999; 2006). The sand has minimal microbial community and hence the demand for oxygen is primarily due to root activity. There was no evidence of anoxic conditions in the wet sand at the bottom of the tube at harvest (Coelho Filho et al. 2013).

The nutrient solution composition was 2.0 mM Ca(NO_3_)_2_, 1 mM KH_2_PO_4_, 4.0 mM KCl, 2.0 mM MgSO_4_, 4.0 mM CaCl_2_.2H_2_O, with the following micronutrients: 60 μM Si, 50 μM B, 50 μM Fe, 15 μM Mn, 0.8 μM Zn, 0.3 μM Cu and 0.1 μM Mo. Sand and nutrient solution were poured into tubes simultaneously so that the sand always fell into liquid to prevent the formation of air pockets in the sand profile. The water table height was maintained at 30 cm below the surface of the sand.

Germinated wheat seeds, with roots all shorter than 1 cm, were transplanted into the sand through a small hole in the centre of the weight or foam at the depth of 2 cm, one seedling per core (Coelho Filho et al. 2013) (Fig. 1). All experiments were carried out in a controlled environment (CE) room with day/night temperatures of 22 and 18 °C, respectively, and a 14 h day length. The relative air humidity was 70 % during the day and 80 % at night. Lighting was supplied by fluorescent tubes, with supplementary tungsten lighting, and the photosynthetic photon flux density was 450 μmol m^−2^ s^−1^ at plant height. Plants were grown for 40 days.

### Plant measurements

During the experiment, daily measurements were taken of the lengths and widths of the first 8-9 leaves as these appeared, using a Perspex ruler. Leaf area was calculated as length x width x 0.73, as used for maize (Mckee 1964), although the value of the coefficient is not crucial when comparing treatment effects.

At harvest the number of tillers and primary root axes were counted, and the length of the longest root was measured. The roots were gently washed out of the sand core system (Coelho Filho et al. 2013). The location of the hole in the basket where each primary root emerged was recorded and the angular spread of roots was calculated by assuming that roots grew in a straight line. Vertical roots and horizontal roots had an angular spread of 0° and 90° respectively. Root angle was defined as the angle between the vertical line and the direct connection between the seed and the point at which a root penetrated the mesh when the plant was harvested. This approach is similar to that used by Oyanagi et al. (1992), although they grew wheat roots in either agar or vermiculite. It was not possible to take account of primary roots that first followed a straight line and then bent downwards. However, when wheat roots are grown against a glass face (e.g. Manschadi et al. 2008), they usually grow in approximately straight lines until they reach an interface (the edge of the rhizobox in the case of Manschadi et al. (2008)), at distances much greater than the dimensions of our hemispherical basket (Fig. 1).

The roots in each sample were washed gently on a fine sieve (0.2 mm mesh size) and spread out on a plastic tray filled with 2–3 mm deep distilled water. Root length and diameter distribution were measured by a scanner running with WinRhizo software (Regent Instruments Inc., Quebec, QC, Canada). Root diameters (d) were recorded in 20 classes between 0 and 3.0 mm, which were bulked into 6 groups: 0 < d < = 0.2, 0.2 < d < = 0.4, 0.4 < d < = 1.0, 1.0 < d < = 2.0, 2.0 < d < = 3.0 and d > = 3.0 mm. The different types of roots (primary and laterals) were not separated. Once root scanning was complete, the shoots and roots were oven dried at 65 °C for 48 h prior to measuring dry weights.

### Experimental treatments and statistical design

The experimental treatments were four wheat cultivars and two levels of impedances. The experiment was conducted in three randomized blocks. Each block contained 8 sand culture pots with all treatment combinations. The complete experiment was replicated twice. The data were analysed with Analysis of Variance using Genstat® V14 (VSN International Ltd, 5 The Waterhouse, Waterhouse Street, Hemel Hempstead, HP1 1ES, UK). The differing numbers for roots on different plants made the root angle data unbalanced and this was analysed with REML (Residual Maximum Likelihood). Data area presented with the standard error of differences, degrees of freedom and *P* values.

Statistical analysis of the leaf elongation measurements was done by modelling the general response as a linear regression and then superimposing the approximate sigmoid shape over time using splines all in the context of REML. This approach was adopted as the exact form of non-linear response over time was not important.

## Results

The two way table of means for shoot dry weight, number of tillers, number of primary roots and maximum root depth is reported in Table 1. There were no significant effects of the interaction between impedance and wheat cultivar on these data, thus the interpretation depends on the main effects. Shoot dry weight of plants with impeded roots was 1.21 g compared to 5.71 g in the non-impeded controls (SED = 0.239, *P* < 0.001 1 df). Root impedance reduced tiller number from 18.7 to 4.0 (*P* < 0.001, 1 df) (Table 1). Cadenza had significantly fewer tillers than the other cultivars; 8.3 tillers for Cadenza compared to 11.7, 11.8 and 13.2 for Avalon, Battalion and Robigus (*P* < 0.001, 3, 34 df SED = 1.039) respectively. There was only a weak effect of the interaction between wheat cultivar and root impedance on tiller number (*P* = 0.079, 3, 34 df) (Table 1). Root impedance decreased the number of primary roots from 42.6 to 16.0 (SED = 1.4, *P* < 0.001, 1 df) (Table 1) and the rooting depth from 11.8 cm to 7.2 cm (SED = 0.76, *P* < 0.001, 1 df) (Table 1).

**Table 1:** The data of shoot dry weight (g), number of tillers, number of primary roots and maximum root depth (cm) for control and impeded roots at the point of harvest

	Shoot Dry weight (g)		Number of tillers		Number of primary roots		Maximum root depth (cm)	
	control	impeded	control	impeded	control	impeded	control	impeded
Avalon	5.91	1.22	19.7	3.8	44.3	14.7	11.2	8.7
Battalion	5.55	1.63	18.3	5.3	39.7	17.8	12.8	6.7
Cadenza	5.65	0.7	14.5	2.2	42.9	13.5	11.9	6.5
Robigus	5.72	1.29	21.8	4.7	43.7	17.8	11.3	6.8
*P*-value								
Treatment	<0.001		<0.001		<0.001		<0.001	
Genotype	0.593		<0.001		0.632		0.828	
Genotype x Treatment	0.474		0.079		0.201		0.371	

The genotype x treatment interaction was also shown. Each value is the mean of four replicates. ANOVA was conducted with *P* Values for treatment (control and impeded), genotype and their interaction (Genotype x Treatment) reported

**Fig. 2:**
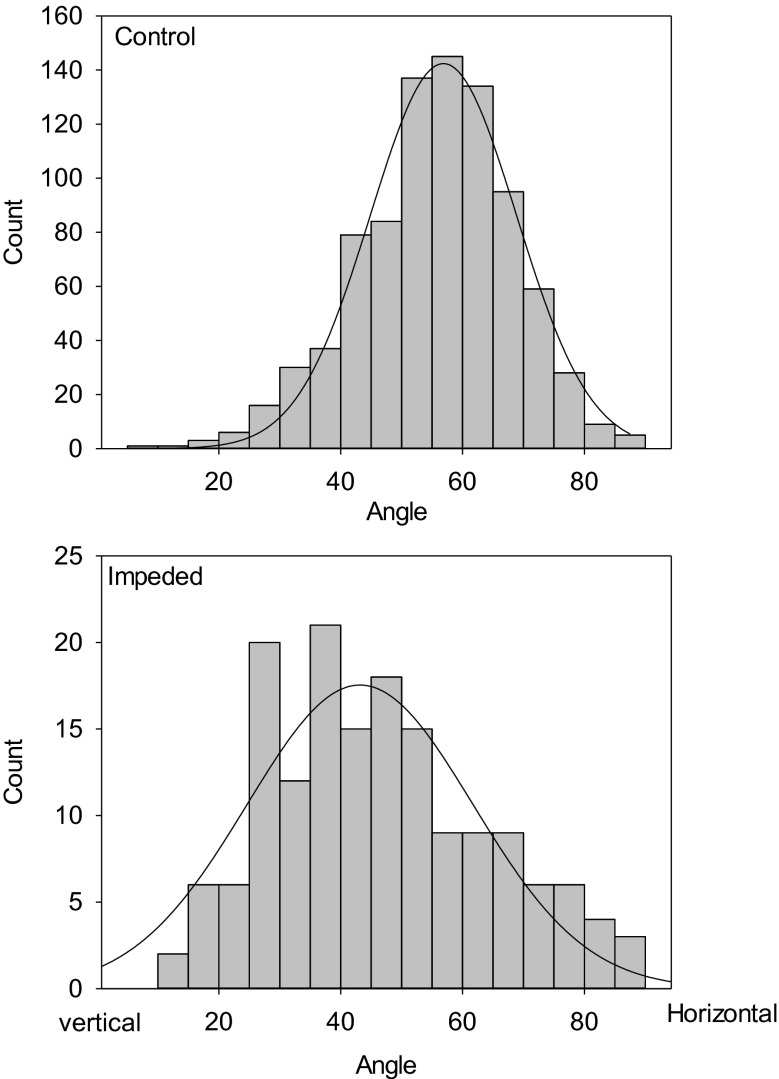
The distribution of root growth angles for non-impeded control roots and impeded roots. Data for all four wheat cultivars were pooled because the interaction between wheat cultivar and impedance was not significant

**Fig. 3:**
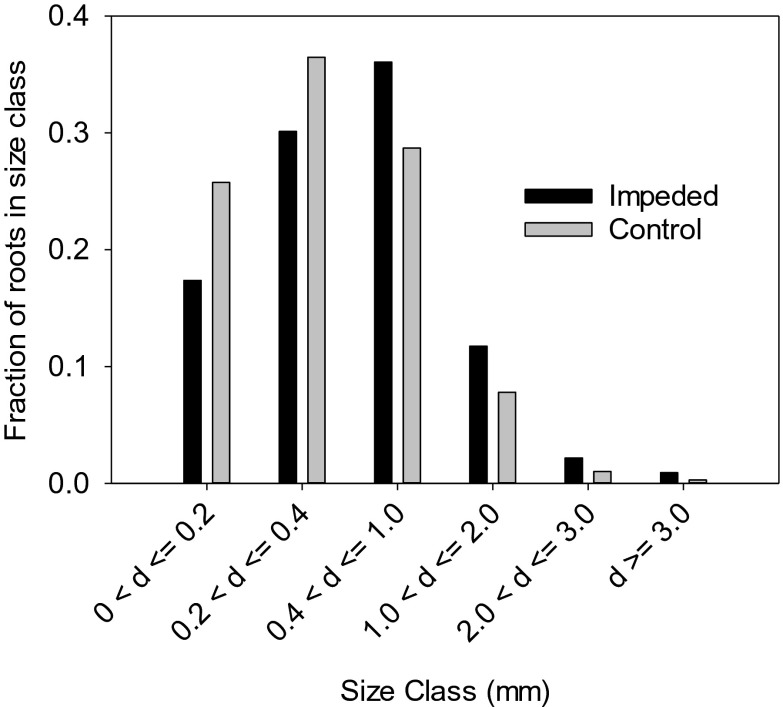
Root diameter size distribution as affected by root impedance. The effect of impedance on root diameter was significant at *P* < 0.001

Impedance significantly (*P* = 0.002, 1 df) decreased root growth angle from 55° to 43° (from the vertical), with impedance causing steeper roots. There was no effect of wheat cultivar on growth angle, but there was a weak interaction with root impedance (*P* = 0.057, 3 df) shown in Table 2. Impedance to root elongation increases the spread of growth angles as well as increasing the mean steepness of root growth (Fig. 2). The distributions of root diameter for the control and impeded roots are shown in Fig. 3. ANOVA showed the interaction between impedance level and root size class was significant at *P* <0.001 (df 5, 95) although root impedance results in thicker roots. There was no effect of cultivar on the root diameter distributions plotted in Fig. 3 (*P* = 0.389).

**Table 2:** Mean angular spread of roots for control and impeded roots

Wheat	Control	Impeded
Avalon	57	43
Battalion	50	46
Cadenza	55	53
Robigus	57	32

The genotype x impedance interaction is significant at *P* = 0.057. An angle of 0° corresponds to a vertical root. The SED is 5.6°. The mean angular spread of roots for control and impeded roots was 55^O^ and 43^O^ (*P* = 0.002) respectively. There was no effect of cultivar (*P* = 0.302)

**Fig. 4:**
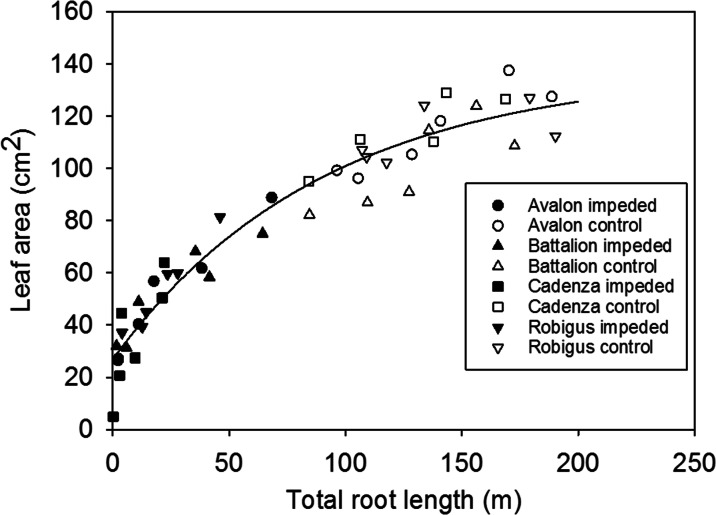
Leaf area plotted against root length. The data were fitted to the curve $Leaf\; area=A+B{R}^{Total\; root\; length}$. A common curve could be fitted to all data which accounted for 93.5 % of the variance (*P* < 0.001) with the following parameter values: *A* = 137.80 (+/- 9.59), *B* = ­110.99 (+/-8.57) and *R* = 0.98904 (+/- 0.00217). Each point is an individual plant.

Over the two experiments, there was a strong relationship between total root length and leaf area (Fig. 4) which explained 93.5 percent of the variance. Root dry matter explained 83.7 % of the variance in shoot dry matter (P < 0.001) with a linear relationship (Shoot dry weight (g) = 2.97 (+/-0.11) Root dry weight (g)).

**Fig. 5:**
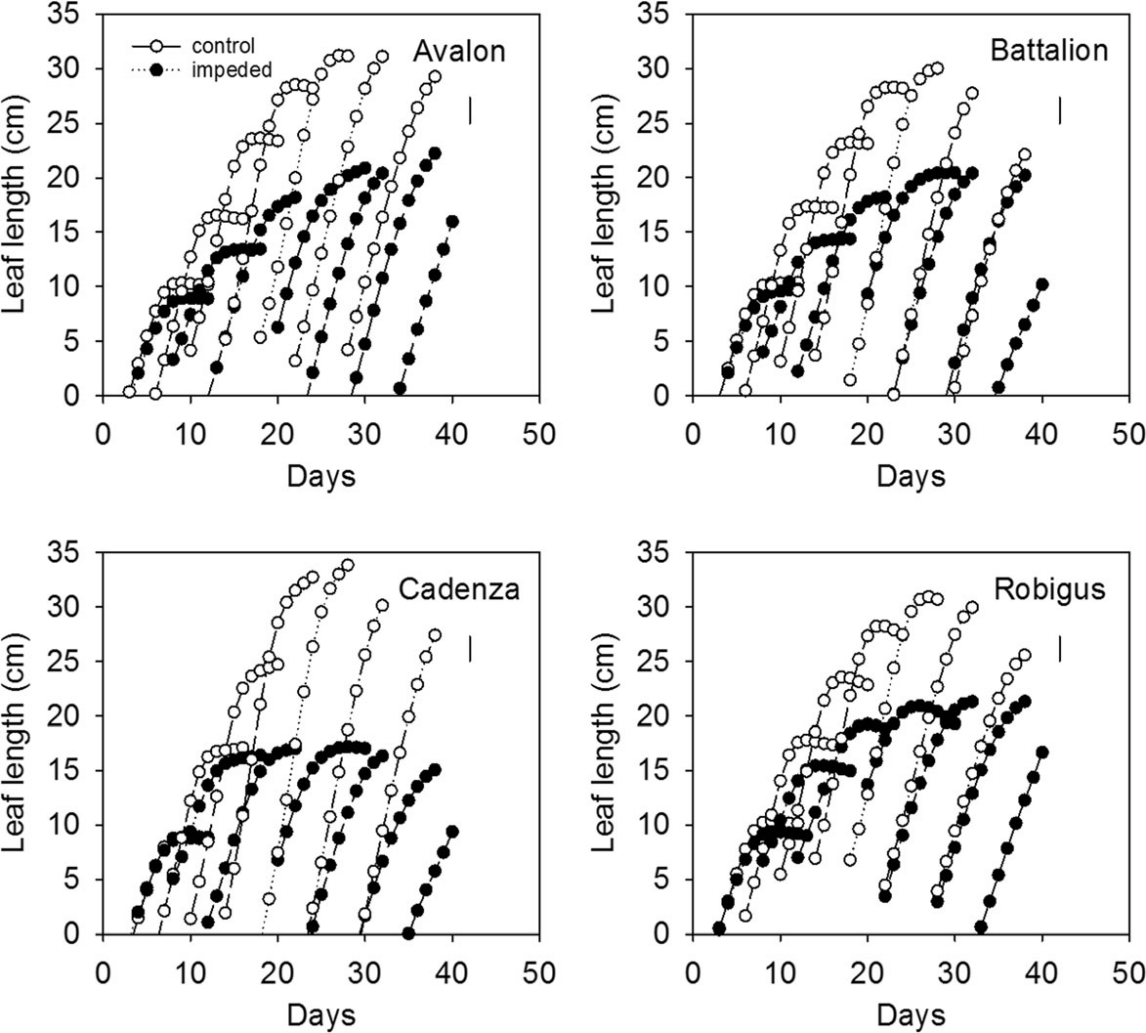
Leaf elongation in four cultivars with impeded roots compared with roots in mechanically weak sand (control). Data are means for the two experiments estimated by fitting a spline function to the data from both experiments. The standard error of differences (SED) from REML analysis is shown. The interaction between cultivar, leaf number, time was significant at *P* < 0.001 (18 df) and the main effect of root impedance and cultivar had a significant effect at *P* < 0.001

To combine leaf length data from the two separate experiments, splines were fitted to estimate leaf length against time for the different leaf numbers over three replicates which were repeated in time (*n* = 6). There was a statistically significant four term interaction between variety, leaf number, root impedance and days of growth (F_18,1315_ = 14.83, *P* < 0.001) (Fig. 5). This interaction represents separate slopes and intercepts for the combinations of the treatment factors: variety, leaf number and root impedance. In addition to this, there were included separate spline terms for individual leaves and root impedance within leaves. In other words, the shapes of the response were different for the various combinations of leaves and root impedance (Chisquare on 1 df = 1020, *P* < 0.001). This effect was particularly marked on leaves 5 to 6. Cadenza leaves were more severely stunted than the other cultivars studied but they had the longest leaves when the roots were not impeded (Table 3). For Avalon, but not the other cultivars, mechanical impedance delayed leaf emergence.

**Table 3:** The final leaf length of leaves 3, 4, 5 and 6

Impedance	Wheat	Leaf length (cm)			
		Leaf 3	Leaf 4	Leaf 5	Leaf 6
Control	Avalon	23.30	27.99	30.39	31.00
	Battalion	23.30	28.46	30.08	29.57
	Cadenza	25.16	33.28	35.39	33.23
	Robigus	22.47	26.83	29.24	28.88
Impeded	Avalon	18.30	19.04	18.55	17.97
	Battalion	18.06	19.67	18.77	15.36
	Cadenza	16.20	15.26	13.84	12.24
	Robigus	18.40	20.02	20.37	19.08

The SED to compare any two of the means in this table is 2.32 cm (*P* < 0.001, 18 df)

## Discussion

### Root growth

We report the novel finding that increased mechanical impedance alters the angular spread of wheat roots (Table 2). The most likely explanation is that root impedance alters the strength of the geotropic effect. Oyanagi et al. (1992) considered two Japanese wheat cultivars (Minaminokomugi and Norin 58). Norin 58 was insensitive to soil water potential, but had relatively steep roots, with mean growth angle of approximately 20° using our convention (0° = vertical root). In contrast, Minaminokomugi had a mean growth angle of 80° in wet conditions but grew steeply (40° to the vertical) at water potentials smaller than -50kPa. In the four cultivars considered (Avalon, Cadenza, Robigus and Battalion), there was only weak evidence that they differed in their response of root angle to soil impedance (Table 2, *P* = 0.057). However, from experiments in gel chambers, it is known that genotypic variability in the angular spread of barley roots exists in young seedlings (Bengough et al. 2004) and in wheat grown in soil-filled rhizotrons (Manschadi et al. 2006). Our data demonstrate an environmental trigger to the direction of root elongation related to mechanical strength of the soil.

Hamada et al. (2012) identified QTLs of root growth angle in the seminal roots of wheat. The explanation as to why impedance or water stress alters the strength of the geotropic response presumably involves the mechano-sensory system in the root tip (Staehelin et al. 2000; Bastien et al. 2013; Toyota and Gilroy 2013). Root impedance has the well-reported effect of increasing root diameter (Fig. 3) (Clark et al. 2008) and it is possible that the altered shape of root tip cells (Bengough et al. 1997) may affect the sedimentation of the starch statoliths, and how they trigger auxin flow. Impedance had the effect of increasing root diameter (Fig. 3), but there was no interaction with cultivar. It is interesting that while water stress per se can reduce root diameter (Sharp et al. 1988) and mechanical impedance causes root thickening (Clark et al. 2008), both water stress and mechanical impedance can cause roots to grow at steeper angles. The angular spread of roots in wheat and other cereals is an important trait associated with adaption to water-limited environments (Manschadi et al. 2006). Our data suggested that there may be a degree of plasticity in this trait and in wheat cultivars we studied the effect of environment was greater than any genetic effect. The possibility that root growth angles are in part determined by the environment (e.g. Table 2), may be an important contributor to root plasticity, allowing exploration of surface nutrient rich layers when the soil is wet and mechanically weak, but favouring steeper root growth to depth in dry and hence stronger layers.

Impedance resulted in shorter roots (7.2 cm compared to 11.8 cm) which is consistent with previous reports using the same sand culture system (Coelho Filho et al. 2013; Whalley et al. 2006). The position of the capillary fringe in our experiments (Fig. 1) may have restricted rooting depth, which can be much greater when roots are grown in unsaturated soil (Jin et al. 2015; Manschadi et al. 2006, 2008). Wheat root growth is greatly affected by the presence of a water table, and a shallow water table (approximately 60 cm deep) limited root growth below 40 cm at 38 days after sowing (Zuo et al. 2006). The presence of a water table is an inevitable consequence of using a sand culture system, as described here and as used elsewhere (Chapman et al. 2011; Clark et al. 2002). Thus with the exception of plants with small root systems, such as Arabidopsis (Chapman et al. 2011), it is probably inadvisable to draw general inferences about rooting depth data obtained in sand culture systems. In comparable sand culture experiments measurements of oxygen diffusion suggest that this is not limiting (Whalley et al. 1999). In future experiments it may be instructive to investigate the effects of supplemental oxygen with a approach comparable to that described for hydroponic systems by Verslues et al. (1998).

### Shoot growth

The impedance to the roots applied in the sand culture system replicates the effects of leaf stunting and lower tiller number observed in field grown wheat (Atwell 1990). Root impedance decreased leaf elongation (Fig. 5) (Whalley et al. 2006) and reduced early shoot and root growth of all genotypes. Our data suggests that the strength of stunting may be related to the *Rht* allele. Cadenza, which has a tall *Rht* allele, had longer leaves in weak control soil, but the effect of root impedance on leaf length was more severe than for the other cultivars (Avalon, Battalion and Robigus) which all contain semi dwarf alleles. This is consistent with our previous work (Coelho Filho et al. 2013) comparing tall, semi-dwarf and dwarfs NILs in a Mercia background, where the sensitivity of leaf elongation to root impedance decreased with the strength of the dwarfing. One of the effects of semi-dwarfing alleles is to reduce leaf length, which is compensated for by a higher photosynthetic rate (Flintham et al. 1997). Under optimal environments, semi-dwarf wheat cultivars tend to out-yield tall wheat cultivars, but tall wheat cultivars are reportedly more resilient to the effects of adverse soil conditions (water limited or nutrient poor soils) (Butler et al. 2005). A greater understanding of the more sensitive leaf stunting response in wheat cultivars with tall *Rht* alleles, may provide some insights into why semi-dwarf wheat cultivars are more sensitive (and tall wheat cultivars less sensitive) to adverse growth conditions.

The number of tillers in Cadenza was smaller than in the other semi-dwarf wheat cultivars considered (Avalon, Battalion and Robigus), consistent with our previous work (Coelho Filho et al. 2013) where tiller number in *Rht* NILs increased in the order tall < semi-dwarf < dwarf; the tall NIL had the fewest tillers. Although root impedance reduced the number of tillers, we found no effect of the interaction between root impedance and cultivar on tiller number. This suggested that the effects of the *Rht* allele, or any other genetic differences between these cultivars, are not implicated in the decreased tiller number in response to root impedance.

### Coordination of root and shoot growth

The growth of all the wheat cultivars was broadly consistent with previously published data (Masle and Passioura 1987; Atwell 1990; Beemster and Masle 1996; Whalley et al. 2006) in that root impedance decreased the size of the shoot and root system. There was a strong relationship between root and shoot growth, best described by that between leaf area and total root length (Fig. 4), which applied across all cultivars. We speculate that this is not due to increased water / nutrient acquisition as these were supplied in abundance, but instead resulting from synthesis of growth hormones in the roots and their transport to the shoot (Dodd 2005). Improved resilience of yield to strong soil may be related the degree of leaf stunting which depends on cultivar (Cadenza was the most sensitive) and sensitivity of tiller number which in this study was only affected by impedance.

## Conclusions

Root impedance increased the steepness of root angular spread via a mechanism that is presently unknown. Root impedance caused a more severe stunting effect on shoot growth of Cadenza compared with Avalon, Battalion or Robigus. Cadenza has a tall *Rht* allele and our finding is consistent with previous accounts of leaf stunting in tall, semi-dwarf and dwarf NILs. This is the first time that genotypic variation in the sensitivity of leaf elongation to root impedance has been demonstrated in commercial wheat cultivars.
